# 16S rRNA gene sequencing reveals an altered composition of the gut microbiota in chickens infected with a nephropathogenic infectious bronchitis virus

**DOI:** 10.1038/s41598-020-60564-8

**Published:** 2020-02-26

**Authors:** Puzhi Xu, Yan Shi, Ping Liu, Yitian Yang, Changming Zhou, Guyue Li, Junrong Luo, Caiying Zhang, Huabin Cao, Guoliang Hu, Xiaoquan Guo

**Affiliations:** 1 Jiangxi Provincial Key Laboratory for Animal Health, College of Animal Science and Technology, Jiangxi Agricultural University, Nanchang, Jiangxi China; 2 School of Computer and Information Engineering, Jiangxi Agricultural University, Nanchang, Jiangxi China

**Keywords:** Biotechnology, Microbiology

## Abstract

Infectious bronchitis virus (IBV), a member of the Coronaviridae family, causes serious losses to the poultry industry. Intestinal microbiota play an important role in chicken health and contribute to the defence against colonization by invading pathogens. The aim of this study was to investigate the link between the intestinal microbiome and nephropathogenic IBV (NIBV) infection. Initially, chickens were randomly distributed into 2 groups: the normal group (INC) and the infected group (IIBV). The ilea were collected for morphological assessment, and the ileal contents were collected for 16S rRNA gene sequencing analysis. The results of the IIBV group analyses showed a significant decrease in the ratio of villus height to crypt depth (P < 0.05), while the goblet cells increased compared to those in the INC group. Furthermore, the microbial diversity in the ilea decreased and overrepresentation of *Enterobacteriaceae* and underrepresentation of *Chloroplast* and *Clostridia* was found in the NIBV-infected chickens. In conclusion, these results showed that the significant separation of the two groups and the characterization of the gut microbiome profiles of the chickens with NIBV infection may provide valuable information and promising biomarkers for the diagnosis of this disease.

## Introduction

Based on the revolution in our understanding of host-microbial interactions in the past two decades, it has been recognized that the gut microbiome is exceedingly complex^1^. We have at least 100 trillion (10^14^) microbial bacteria and a quadrillion viruses in and on us^2^. Collectively, the microbial bacteria that are located in and on the body make up our microbiota, and the genes they encode are recognized as our microbiome. Numerous studies have shown that the role of intestinal flora includes nutrient absorption, mucosal barrier fortification, xenobiotic metabolism, angiogenesis, immunity, and response to infection^3–5^. In human studies, many diseases are closely related to anomalies of the intestinal microbial community, comprising of liver cirrhosis, allergies, obesity, diabetes and so on^6–8^. Indeed, indigenous microbiota play a crucial part in the prevention and therapy of microbial infections and are frequently referred to as a “forgotten organ”^1,9,10^. Our recognition of the gut microbiota and the identification of its composition when contrasted between diseased and healthy states allow the determination of dysbacteriosis, which may be associated with disease progression; thus, this may serve as a new way to diagnose, prevent and treat interventions^11^.

Although the ileum microbiota is comparatively stable under normal conditions, it is readily affected by various diseases^12,13^. The gut dysbacteriosis induced by viruses might promote the replication and transmission of viruses in organisms; this phenomenon has been found for simian immunodeficiency virus (SIV) and human immunodeficiency virus (HIV)^14,15^. Infectious bronchitis virus (IBV), which can cause a highly infectious respiratory disease and urogenital illness characterized by gout or nephritis in chickens, is a gamma coronavirus in the family Coronaviridae^16,17^. All IBV strains can infect a wide range of chicken epithelial surfaces, such as the trachea, kidney, proventriculus and intestines^18^. Some IBV strains are called nephropathogenic IBV (NIBV) because they lead to severe kidney infections in addition to respiratory infections. It is noteworthy that IBV was detectable in intestinal epithelial cells^19^. Many studies point out that the chicken ileum is a significant location for digestion and nutrient absorption and is home to a diverse microbial community^20,21^. However, there has been little exploration of the alteration in the gut microbial community structure and abundance in chickens with IBV infection. Thus, there is an urgent need to establish the aetiological link between the IBV and gut microbiota. Here we investigated the ileum microbiome in chickens infected with IBV for a more detailed and comprehensive evaluation.

The exploration of the chicken intestinal microbiota has primarily been researched by culture-based methods^22^. However, these methods have limitations: they are inapplicable to nonculturable bacteria and are selective for readily cultivated bacteria^13^. To address these limitations, molecular techniques have been used to characterize the intestinal microbiota. Illumina sequencing of single or multiple hypervariable region amplification in the 16S rRNA gene is an effective approach for gut microbiome analysis^23^. In the present study, we elucidated the effects of NIBV infection on the chicken gut microbiome by 16S rRNA gene sequencing. The relative abundance data from the ileum microbiome suggested differences between chickens in the INC group and those in the IIBV group infected with NIBV. Therefore, further investigation into the mechanism of this shift will help us understand IBV infection and provide a potential approach to diagnose, treat and prevent interventions.

## Results

### Viral load and histopathology

**Figure 1:**
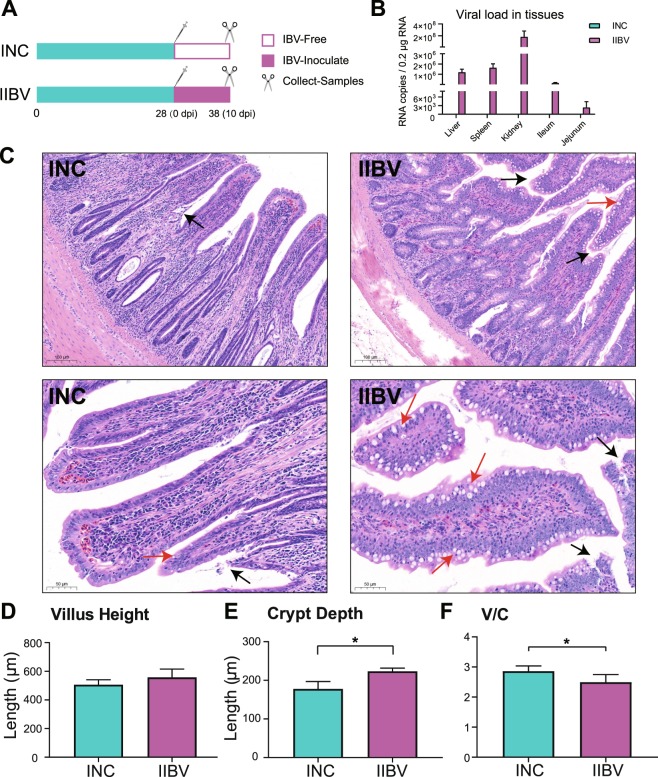
Changes in chickens with SX9 infection. (**A**) Experimental design, including viral inoculation time and sample collection time. (**B**) Viral load in different chicken tissues quantified by RT-qPCR (n = 6). (**C**) Histopathological changes in the ilea of chickens challenged with the NIBV strain SX9 [H&E staining]. The black arrow shows the shed villus epithelial tissue, and the red arrow shows the goblet cells (vacuole). The villus height (**D**) and crypt depth (**E**) and the ratio of the villus height to the crypt depth (V/C) (**F**) of the ilea were measured by Image-Pro Plus 6.0. The data are shown as the means, with SEMs in (**D** to **F**). The asterisk superscripts on the bars indicate significant differences compared with the control group (t test, *P < 0.05).

All the NIBV-infected chickens in the IIBV group were listless, huddled together, had watery faeces, and displayed ruffled feathers (score = 2, according to the methods described by Avellaneda *et al*.^24^) from 3 to 9 days post-inoculation (dpi), and the morbidity was higher than 90%. These clinical symptoms were not observed in the INC group. The quantification of the viral load by RT-qPCR showed values ranging from 2.96 × 10^3^ to 3.84 × 10^8^ genomic copies per 0.2 μg of RNA, with the lowest viral load in the jejuna and the highest in the kidneys (Fig. 1B). It was noted that the viral load in the ilea was much higher than that in the jejuna, reaching 1.39 × 10^5^ genomic copies per 0.2 μg of RNA. No viral RNA was detected in the INC group. There were more goblet cells, which appeared as vacuoles when stained by H&E, in the IIBV group than in the INC group (Fig. 1C, red arrow). In addition, there were many shed villus epithelial tissue that appeared in the ilea of the IIBV group (Fig. 1C, black arrow). Among the ilea, there was no significant difference in villus height (Student’s t test, P = 0.094, Fig. 1D), but crypt depth was significantly increased (Student’s t test, P = 0.0003, Fig. 1E), and the V/C ratio was significantly decreased (Student’s t test, P = 0.016, Fig. 1F).

### Description of the sequencing data

**Figure 2:**
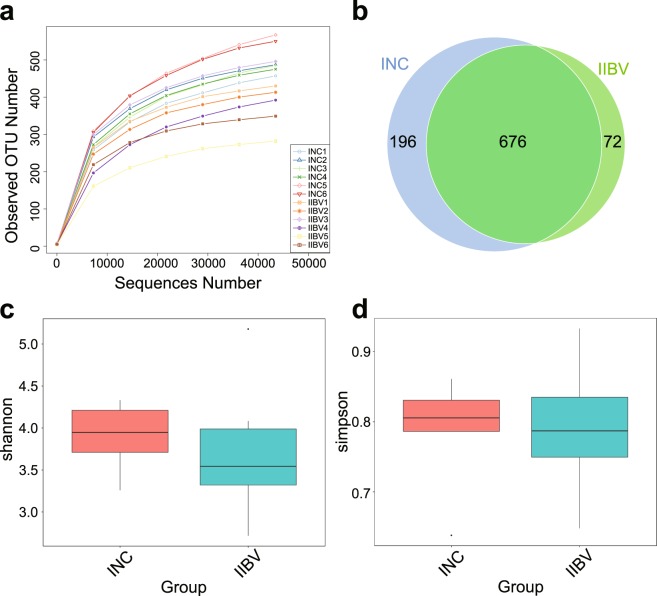
Sample abundance and evaluation of the alpha diversity. (**a**) Rarefaction curves of the OTU number in the INC and IIBV groups (n = 6 per group). The number of sequences sampled represents the number of sequencing reads, which were clustered into operational taxonomic units (OTUs) at 97% similarity. (**b**) Venn diagram showing the overlap in the differential abundance of the OTUs in the control and NIBV-infected chickens. The bacterial diversity in the INC and IIBV groups was estimated by the Shannon index (**c**) and Simpson index (**d**). The results were compared using a Wilcox test.

After two separate runs on an Illumina HiSeq. 2500 platform and quality-filtering as described in the methods, 698,855 total sequences were identified for further analysis. The rarefaction curves (Fig. 2a) showed that the sequencing depth was near saturation and that the sequencing data included most of the 16S rRNA gene information in the samples.

### A decrease in the microbial diversity in the ilea of chickens with NIBV infection

The sample community richness was evaluated based on the operational taxonomic units (OTU)^25^ counts in each single sample as shown in Table 1. As shown by the OTU counts, the number of OTUs in the ilea of the IIBV group chickens was less than that of the INC group chickens (Fig. 2b). According to the lower ACE and Chao1 indices, NIBV infection reduced the community richness compared to that in the INC group (Table 1). In addition, neither the Shannon nor the Simpson indices were significantly different between the IIBV group and the INC group within the experiment (Wilcox, P = 0.5887 and P = 0.9372, respectively). The higher Shannon and Simpson index indicated a higher bacterial diversity, which meant that the NIBV infection decreased the microbial diversity compared to that in the INC group (Fig. 2c,d). In conclusion, these data indicated a smaller bacterial diversity in the ileal microbiota of NIBV infected chickens.

**Table 1:** Number of OTUs per groups and estimate of sequence diversity and richness.

Sample	Reads	OTUs	Shannon	Simpson	Chao1	ACE	Goods Coverage
INC1	69,809	457	3.258	0.638	541.726	536.081	0.998
INC2	67,468	487	4.014	0.786	523.181	537.556	0.998
INC3	63,114	485	3.654	0.824	567.179	585.597	0.997
INC4	59,820	475	3.879	0.787	533.897	536.607	0.998
INC5	69,855	567	4.332	0.833	757.815	718.399	0.996
INC6	61,207	550	4.276	0.861	602.009	620.741	0.998
IIBV1	45,655	430	3.706	0.802	466.849	464.887	0.999
IIBV2	52,163	413	4.083	0.846	452.276	455.064	0.998
IIBV3	55,708	496	5.178	0.933	549.915	557.015	0.998
IIBV4	49,563	392	3.301	0.772	458.944	465.737	0.998
IIBV5	51,018	282	2.717	0.648	296.138	305.69	0.999
IIBV6	53,475	349	3.38	0.742	371.021	373.35	0.999

### NIBV infection altered the ileal bacterial microbiome composition in chickens

**Figure 3:**
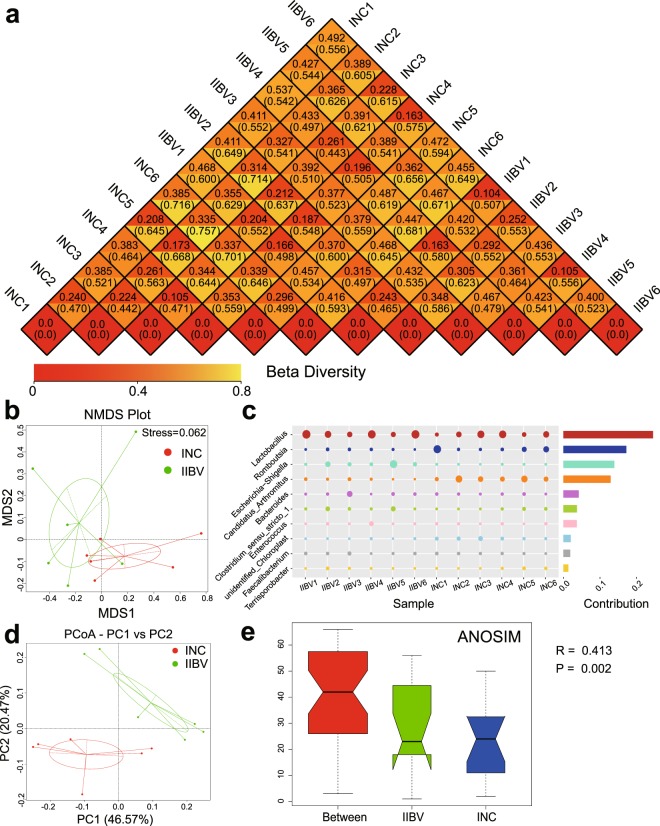
Exploration of the beta diversity in the INC and IIBV groups. (**a**) Beta diversity index heatmap. The number in each square is the difference coefficient between the two samples. The smaller the difference coefficient is, the smaller the difference in species diversity. In the same square, the upper and lower values represent the weighted and unweighted UniFrac distances. (**b**) The nonmetric multidimensional scaling (NMDS) plot, showing the difference in the bacterial communities according to the Bray-Curtis distance. (**c**) Ten species (at the phylum level) with the highest contributions to the difference in the bacterial communities based on Simper analysis and Bray-Curtis distance. (**d**) Principal coordinate analysis (PCoA) plot of the similarities between the different groups derived based on UniFrac distance. Principal components (PCs) 1 and 2 explained 46.57% and 20.47% of the variance, respectively. (**e**) Analysis of similarities (ANOSIM) of the bacterial communities of the ileal samples between the INC and IIBV groups based on Bray-Curtis distance (R = 0.43 > 0 indicates that the differences between the groups are significant, P = 0.002 indicates that the statistics are significant).

To compare the global differences in the bacterial community composition between the NIBV infected chickens and the INC group chickens, the Bray-Curtis similarity and unweighted UniFrac^26^ were calculated (Fig. 3a). We also performed nonmetric multidimensional scaling (NMDS)^27^ of all the samples using count-based Bray-Curtis similarity at the OTU level to explore the differences in the bacterial community composition among the two main groups of chickens^28^. As shown in Fig. 3b, the results provided good discrimination between the groups, suggesting that the bacterial community composition of the IIBV group was different from that of the INC group. Simper (similarity percentage) is a decomposition of the Bray-Curtis difference index, which quantifies the contribution of each species to the difference between the two groups. The results showed the top 10 species in the genus level and their contributions to the differences between the two groups (Fig. 3c).

We utilized principal coordinate analysis (PCoA) to visualize visualizing the similarity of the microbial community structures and the phylogenetic distances between the samples by using the phylogenetic-tree-based weighted UniFrac metric. Figure 3d is a UniFrac PCoA-based comparison of the microbial community from the ilea, showing that the NIBV-infected chickens and the normal chickens had a clear separation. In addition, analysis of similarity (ANOSIM) based on the Bray-Curtis distance (R-value = 0.413, P = 0.002, Fig. 3e) and analysis of molecular variance^29^ based on the weighted UniFrac distance (P-value = 0.027, AMOVA) showed a significant separation of the two groups. These results suggested distinct differences in the bacterial composition between the INC group and IIBV group.

**Figure 4:**
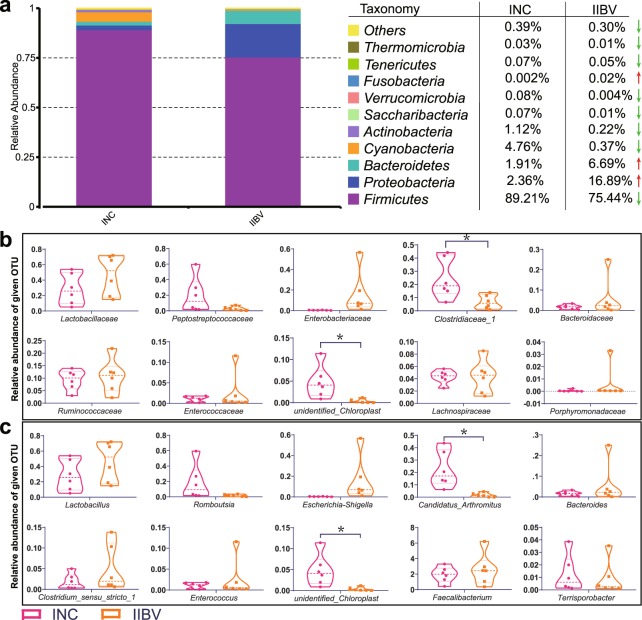
Aggregate microbiota composition at different levels in the INC and IIBV groups. (**a**) Bar plot of the identified bacterial phylum in the analysed samples. The legend reports the average of the relative abundance of each phylum in both animal groups. The abundance of bacteria is shown at the family (**b**) and genus (**c**) levels. Only the families and genera with the top 10 highest abundances were plotted (t test, *P < 0.05).

An inspection of the predicted taxonomic profiles at the phylum level for all the samples indicated that *Firmicutes* (82.3%) was the major phylum of the ileal community, exceeding *Proteobacteria* (9.6%) and *Bacteroidetes* (4.3%). Notably, at the phylum level, the abundance of *Firmicutes* decreased (from 89.21% to 75.44%) in the IIBV group compared to that in the INC group, whereas there was an increase in the abundances of *Proteobacteria* (from 2.36% to 16.89%) and *Bacteroidetes* (from 1.91% to 6.69%) (Fig. 4a). Evidently, *Lactobacillaceae*, belonging to the phylum *Firmicutes*, was the most abundant family in the ileum. The family *Clostridiaceae 1* had significantly lower abundance in the IIBV group than in the INC group (P < 0.05) (Fig. 4b). *Lactobacillus*, belonging to the family *Lactobacillaceae*, was the most abundant genus in the ilea. The genus *Candidatus Arthromitus*, belonging to the phylum *Firmicutes*, and the genus *unidentified Chloroplast*, belonging to the phylum *Cyanobacteria*, were significantly less abundant in the IIBV group than in the INC group (P < 0.05) (Fig. 4c).

**Figure 5:**
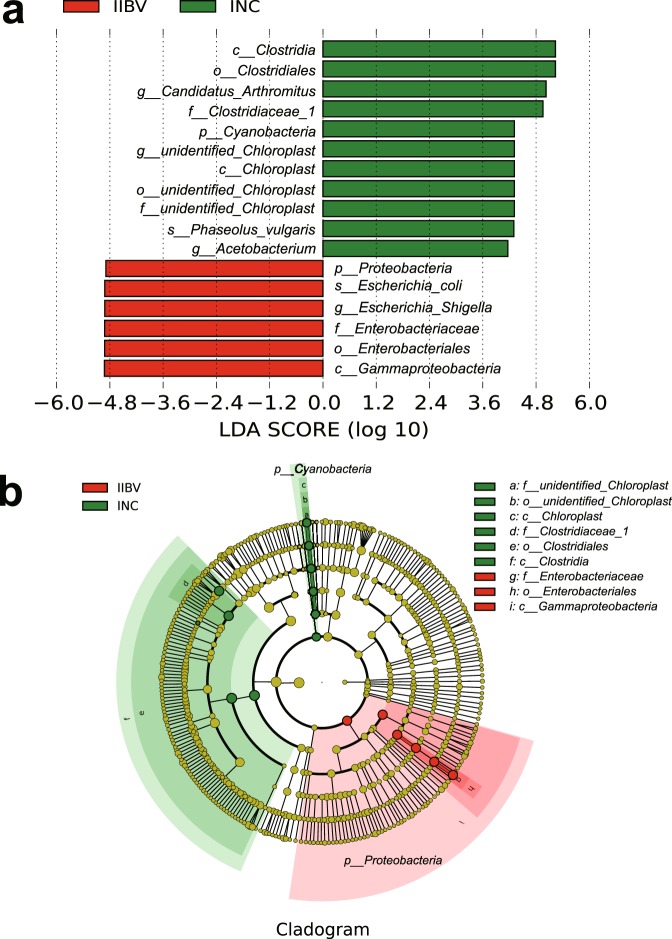
Different structures of gut microbiota in the INC and IIBV groups, according to the LEfSE analysis. (**a**) Taxonomic biomarkers found in the INC (green) and IIBV (red) groups by linear discriminant analysis effect size (LEfSE). (**b**) Cladogram plot of the biomarkers. The size of the node represents the abundance of the taxa. Only taxa with LDA scores (log 10) >4 are shown.

The linear discriminant analysis effect size (LEfSe) analysis was performed in this study to identify distinctive features between the two groups^30^. The differentially abundant phyla detected showed that the *Cyanobacteria* phylum was predominant in the INC group, while the most abundant phylum in the IIBV group was *Proteobacteria* (Fig. 5a). For the IBV-infected chickens, there was an overrepresentation of *Enterobacteriaceae* and underrepresentation of *Chloroplast* and *Clostridia* (Fig. 5b).

### Predicted metagenomic functions showed the effects of the NIBV infection

**Figure 6:**
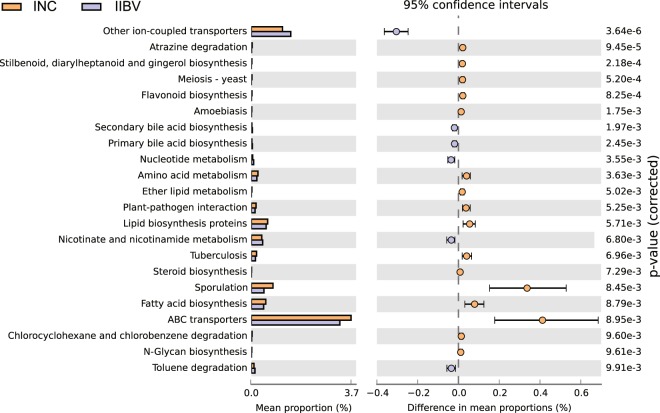
Predicted differential KEGG pathways in the INC and IIBV groups. The PICRUSt-predicted relative abundance of the KEGG pathways (KEGG level 3) was compared between the INC and IIBV groups. The statistical analysis was conducted using White’s nonparametric t-test between the two groups, and only the significant differences in the KEGG pathways (with a P-value < 0.01) are shown.

To gain insight into the relationship between NIBV infection and gut microbiome functions, Phylogenetic Investigation of Communities by Reconstruction of Unobserved States (PICRUSt)^31^ was implemented to predict the potential metagenomes from the community profiles of the normalized 16S rRNA genes (Fig. 6). The results showed that there were sixteen pathways from level 3 of the Kyoto Encyclopaedia of Genes and Genomes (KEGG, http://www.genome.jp/kegg/) enriched in the INC group (*P* < 0.01, White’s non-parametric t-test): atrazine degradation; stilbenoid, diarylheptanoid and gingerol biosynthesis; meiosis-yeast; flavonoid biosynthesis; amoebiasis; amino acid metabolism; ether lipid metabolism; plant-pathogen interaction; lipid biosynthesis proteins; tuberculosis; steroid biosynthesis; sporulation; fatty acid biosynthesis; ABC transporters; chlorocyclohexane and chlorobenzene degradation; and N-glycan biosynthesis. Six pathways were enriched in the IIBV group: other ion-coupled transporters; secondary bile acid biosynthesis; primary bile acid biosynthesis; nucleotide metabolism; nicotinate and nicotinamide metabolism; and toluene degradation (Fig. 5). Altogether, these data suggest that NIBV infection alters the metabolic functions of ileal microbiota and therefore deserves further investigation.

## Discussion

The gut microbiome contains very diverse bacteria and influences myriad host functions^28^. Under pathological conditions, the microbial community composition changes, including an increase and decrease in bacterial populations, causing changes in a wide rage of metabolic functions^32,33^. Accordingly, it is certainly necessary to understand the bacterial community composition and changes in gut microbiota with IBV infection. However, the composition of the gut microbiota and how the coronavirus can affect the gut microbiota are still unclear. Therefore, the effects of IBV infection on chicken ileum damage and the ileum microbiome were investigated in our study by using 16S rRNA gene sequencing. 16S rRNA gene sequencing has been applied extensively for assessing the phylogenetic distribution of metagenomes. It is known that the bacterial 16S rRNA gene consists of nine hypervariable regions, and sequences generated by applying different combinations of these regions generally present differing profiles of microbial diversity. Therefore, the optimal choice of the hypervariable region(s) and primer combination vary between various ecological communities. Previous research proved that the highest bacterial diversity and species richness in chicken gut microbiomes were obtained using the primer set corresponding to the V3-V4 region^34,35^. Based on these investigations, in our study, we selected primers targeting the V3-V4 regions of the 16S rRNA gene.

The complete intestinal structure is essential for digestive and absorptive functions which are intimately associated with the morphological transformations in villus length and crypt depth^36,37^. In the current study, NIBV infection increased the crypt depth and lowered the ratio of villus height to crypt depth in the ileum compared to those in the INC group. These findings suggest that NIBV infection can destroy intestinal mucosal integrity and may slow the growth of intestinal villus epithelial cells. Furthermore, it was reported that intestinal goblet cells play a crucial defensive role in the intestine by synthesizing and secreting several mediators, including mucins and trefoil factor family peptides, which serve defensive and healing functions in the gut^38–40^. In this study, NIBV infection obviously increased the number of goblet cells compared to that in the normal group, which indicated hyperplasia of the goblet cells in the ileum during NIBV infection and may be associated with microbial challenges.

Higher diversity and integrity of gut microbiota is favourable to the intestinal ecosystem^41,42^. According to recent research, viral infection can lower microbial diversity in the gut microbiota according to recent research^43–45^. Consistent with previous findings, in this study, our results demonstrated that NIBV infection decreases the diversity and richness of gut microbiota (Fig. 2c and Table 1). The analysis of the taxonomic composition displayed a reduction in the *Firmicutes* and *Cyanobacteria* phyla and an enrichment of the phylum *Proteobacteria* and *Bacteroidetes* in the ilea. Importantly, the relative abundance of *Enterobacteriaceae*, particularly *E. coli*, was increased in the ilea of NIBV-infected chickens. Moreover, the LEfSe results showed that *Enterobacteriaceae* belonging to the phylum *Proteobacteria* were over-represented in the IIBV group. Previous research shows that in the case of intestinal inflammation, epithelial cells reduce their capacity to undergo beta-oxidation, with the consequence of an increased availability of oxygen, which is thought to promote the bloom of *Proteobacteria* and the dysbiosis of the gut microbiota^46,47^. Further investigations acknowledged that nitrate produced by the host during inflammatory conditions can be exploited by commensal *Enterobacteriaceae*, which thus become predominant^48^. Therefore, the increased enrichment of *Proteobacteria* revealed that the intestinal inflammation in the ilea of NIBV-infected chickens represent a “microbial signature” of NIBV infection. The results also showed that NIBV infection decreased the abundance of the members of the *Cyanobacteria* in the ilea compared to that in the INC group chickens. The *Cyanobacteria* group has great biodiversity due to its secondary metabolites, which show a broad variety of biological activities, including antibacterial, antiviral, anticancer and immunomodulator or protease inhibitor activities^28,49,50^. Our results showed a reduction in *Cyanobacteria*, which indicated that the ability of the chicken ileum to clear viruses was weakened by NIBV infection. Clearly, in the case of viral infection, changes in general metabolic function are closely related to the gain and loss of microbial populations.

Multiple studies suggest that gut microbiota influences the host metabolism^51–53^. Cox *et al*. showed that disruption of microbiota (reduction in *Allobaculum*, *Lactobacillus*, *Rikenellaceae* and *Candidatus Arthromitus* (segmented filamentous bacteria, SFB^54^)) can induce obesity^55^. In the present study, we found that *Candidatus Arthromitus* was dramatically reduced in the IIBV group compared to that in the INC group. However, due to the lack of records of body weight and body fat percentage (BFP) data, it was difficult to verify the direct effect of the reduction in the abundance of *Firmicutes*, such as *Candidatus Arthromitus*, on the body in this experiment. Fortunately, in the present study, we predicted the unobserved character states in the bacterial community by using PICRUSt, which is generally applied to investigate animal intestinal function^56^. With respect to the PICRUSt results, the “primary bile acid biosynthesis” and “secondary bile acid biosynthesis” pathways were significantly enriched in the ilea of the IIBV group chickens. Bile acids are cholesterol-derived compounds synthesized in the liver, and gut microbiota can transfer primary bile acids in the small intestine via deconjugation, dehydroxylation, or dehydrogenation to form so-called secondary bile acids, which promote the intestinal absorption of lipids and affect energy metabolism mainly via the farnesoid X receptor (FXR) and G protein-coupled receptor (TGR5)^57–59^. A great number of observational studies have indicated that meta-inflammatory disorders, such as obesity and type 2 diabetes (T2D), are always associated with an increase in total bile acid (BA) concentrations^60,61^. In addition, Annika *et al*. showed that the levels of iso-DCA, which is a common BA produced by bacteria, were significantly increased in humanized mice with low *Clostridia* abundance. As highlighted above, we know that the enrichment of bile acid biosynthesis is closely related to the loss of *Clostridia*, which may act as an important part of host metabolism in response to NIBV infections.

In conclusion, existing literature highlights the vital role of gut microbiota in the defence against viral infection. Our results suggested that relative abundance data from the ileal microbiota may differentiate the INC group chickens from the IIBV group chickens infected with NIBV. Microbiome profiles could be a biological indicator of IBV infection; therefore, further investigation into the mechanism of this shift will help us understand IBV infection and provide a new approach to diagnose, prevent and treat infections.

## Methods

### Viral strains

The IBV strain was isolated in Nanchang, China in 2011 and stored by the College of Animal Science and Technology, Jiangxi Agricultural University and is designated SX9 throughout this study. At the affected farm, the diseased chickens had the same clinical symptoms: the kidney parenchyma of the dead birds was pale, swollen and mottled. These results show that the IBV strain SX9 has strong nephropathogenic characteristics. The IBV strain SX9 used in this study was propagated in 10-day-old specific-pathogen-free (SPF) embryonated eggs (Jinan sais poultry Co., Ltd., Shandong, China) by the allantoic route. The IBV viral titer (median embryo lethal dose, ELD_50_) was evaluated in the 10-day-old SPF eggs and calculated by the Reed-Muench method.

### Experimental design

A total of 240 healthy Hy-Line variety brown chickens were randomly divided into two experimental groups: a normal group (INC) (30 chickens per subgroup) and a diseased group (IIBV) (30 chickens per subgroup). As shown in Fig. 1A, 0.2 ml of 10^5^ ELD_50_ of SX9 was intranasally inoculated into each chicken in the IIBV group at 28 days of age, while 0.2 ml of sterile physiological saline was administered to each chicken in the INC group. Two chickens were randomly chosen per subgroup at 10 dpi and were euthanized by carbon inhalation. The livers, spleens, kidneys, ilea and jejuna were quickly separated from the bodies in a sterile environment. The ilea were isolated for morphological assessment, and ileal content samples were gathered for bacterial V3-V4 16S rRNA gene sequencing. The rest of the animals were euthanized according to the animal care guidelines of Jiangxi Agricultural University.

### Viral load quantification by RT-qPCR

Total RNA was extracted from the livers, spleens, kidneys, ilea and jejuna with RNAiso Plus (Takara, Japan) following the manufacturer’s protocol. The RNA was quantified using a NanoDrop 1000 Spectrophotometer (Thermo Fisher Scientific, USA). The cDNA was prepared from 2 μg of RNA. The QPCR was performed with primers (forward: 5′-CCATGGCAAGCGGTAAAGCAR-3′; reverse: 5′-CCACTCAAAGTTCATTCTCTCC-3′) and a QuantStudio 7 Flex Real-Time PCR system (ABI Thermo Fisher Scientific, USA). In brief, the amplification was performed using 10 μl volume reactions in a 96-well plate format with the following conditions: 94 °C for 30 s, then 36 cycles of 94 °C for 5 s, 60 °C for 30 s and 72 °C for 30 s. The IBV RNA copy levels were quantified by comparison with a standard curve generated using dilutions (6.67 × 10^3^–6.67 × 10^10^ copies) of the IBV N gene containing plasmid. The IBV RNA levels are expressed as IBV genome copies per 0.2 μg of RNA using the standard curve.

### Histopathology

Sections of the ileal tissues were stained with haematoxylin and eosin (H&E) for assessment of the general ileal morphometry. For each slice, fields were randomly selected, from which all villi were quantified by Image-Pro Plus 6.0.

### 16S rRNA gene sequencing

16S rRNA gene sequencing was performed as described previously^62^. In short, the total genomic DNA in the ileal contents was first extracted, and then, using the genomic DNA as a template, the 16S rRNA gene of the V3-V4 region was amplified using specific primers (forward 341 F: CCTAYGGGRBGCASCAG and reverse 806 R: GGACTACNNGGGTATCTAAT) tagged with the unique barcode. Then the desired size (approximately 400–450 bp) of the PCR products was selected to prepare the sequencing libraries, and the index codes were added. Finally, the samples were sequenced on an Illumina HiSeq. 2500 platform.

### Statistical analysis of the data

Samples were created from the paired-end reads by cutting off their unique barcode and primer sequence. The paired-end reads were merged using Fast Length Adjustment of SHort reads (FLASH) software (version 1.2.7, http://ccb.jhu.edu/software/FLASH/)^63^, and raw FASTQ data has been submitted in the SRA database of the NCBI with accession number PRJNA 533742. To obtain high-quality clean tags, quality filtering of the raw tags was performed using the Quantitative Insights Into Microbial Ecology software (QIIME, version 1.9.1, http://qiime.org/scripts/split_libraries_fastq.html)^64^ quality-controlled process. The chimaeric sequences were removed by using the UCHIME Algorithm (http://www.drive5.com/usearch/manual/uchime_algo.html), and then the effective tags were obtained^65^. The sequences were clustered into OTUs at 97% similarity by UPARSE software (version 7.0.1001, http://drive5.com/uparse/)^25^. In addition, the GreenGene database and MUSCLE software (version 3.8.31, http://www.drive5.com/muscle/) were used to annotate the taxonomic information and conduct the multiple sequence alignment, respectively^66^.

The alpha diversity index was calculated: Chao1 and ACE were used to calculate the bacterial community richness and the Shannon and Simpson indices were calculated to reflect the community diversity^67^. The beta diversity index was calculated using UniFrac and Bray-Curtis distances in QIIME to compare and analyse the composition of the bacterial communities in different samples. PCoA was analysed by the “WGCNA”, “stats” and “ggplot2” packages in R. NMDS and ANOSIM were conducted using the “vegan” package, and a simper analysis was performed by the “simper” package. To find the differences in the biomarkers between the uninfected chickens and chickens infected with the NIBV, we performed a LEfSe analysis (http://huttenhower.sph.harvard.edu/lefse/)^30^ with the following prerequisites: the p value was 0.05, and the threshold on the logarithmic linear discriminant analysis (LDA) score was 4.0^30,68^. In addition, a Phylogenetic Investigation of Communities by Reconstruction of Unobserved States (PICRUSt, version 1.0.0, http://picrust.github.com/)^31^ was performed to predict the ileal microbial community function of the two groups of chickens. Finally, a statistical analysis of the taxonomic and functional profiles (STAMP, version 2.1.3, http://kiwi.cs.dal.ca/Software/STAMP)^69^ was used for further exploration of the results that were clustered in level 3 of the KEGG analysis module in the PICRUSt analysis.

### Statistical analysis

GraphPad Prism (La Jolla, CA, USA) was used for statistical analysis and visualization. A P-value of <0.05 was considered statistically significant.

### Ethical approval

The Institutional Animal Care and Use Committee of Jiangxi Agricultural University approved these animal experiments (approval ID: JXAULL-2017003), and all the animal experiments adhered rigorously to the animal care guidelines of Jiangxi Agricultural University.

## Data Availability

The 16S rRNA sequencing data for this study were submitted to the NCBI Sequence Read Archive (SRA) under the BioProject PRJNA 533742.
